# Beyond Adenosine Deaminase (ADA) and Mycobacterial Growth Indicator Tube (MGIT): The Utility of Xpert MTB/RIF Ultra for Diagnosis and Resistance Determination in Suspected Tubercular Pleural Effusion in a High-Burden State

**DOI:** 10.7759/cureus.113551

**Published:** 2026-07-28

**Authors:** Tanya, Ankana Saha, Praveen Kumar Pandey, Subhadeep Saha

**Affiliations:** 1 Respiratory Medicine, Max Super Speciality Hospital, Delhi, IND; 2 Anatomy, University College of Medical Sciences, Delhi, IND

**Keywords:** cbnaat, drug-resistant tuberculosis, eptb, genexpert, genexpert ultra, mgit, tb pleural effusion, tuberculosis

## Abstract

Background: Diagnosis of tubercular pleural effusion (TPE) is difficult and always controversial unless proved microbiologically. Pleural fluid is almost always paucibacillary, leading to low microbiological yield. Physicians often rely on pleural fluid biochemistry and clinical judgment for initiating antitubercular therapy (ATT). Xpert *Mycobacterium tuberculosis*/rifampicin resistance Ultra (Xpert MTB/RIF Ultra) (Cepheid, Sunnyvale, CA) has been designed to ease the diagnostic dilemma and improve sensitivity in paucibacillary tuberculosis (TB).

Objectives: This study aimed to evaluate the diagnostic utility of Xpert MTB/RIF Ultra in suspected TPE and assess its role in rifampicin resistance determination.

Methods: This retrospective observational study was conducted at a tertiary care center in Northern India. Patients with exudative lymphocytic pleural effusion, elevated adenosine deaminase levels, and clinic-radiological suspicion of TB who underwent pleural fluid Xpert MTB/ RIF Ultra and mycobacterial growth indicator tube (MGIT) culture testing were included. Clinical response to ATT and radiological improvement were assessed during follow-up.

Results: Twenty-one patients were included in the final analysis. Xpert MTB/RIF Ultra demonstrated positivity in 14 patients, while MGIT culture remained negative in all cases. All Ultra-positive patients showed significant clinical and radiological improvement following ATT, supporting the diagnosis of pleural TB despite MGIT culture negativity. Most of the Ultra-positive samples demonstrated “Trace” positivity with indeterminate rifampicin resistance results, limiting reliable drug resistance interpretation.

Conclusion: Xpert MTB/RIF Ultra appears to be a useful adjunctive diagnostic tool in suspected TPE, where culture positivity is low. Although rifampicin resistance determination remains challenging in paucibacillary pleural fluid specimens, Ultra may help improve microbiological support for diagnosis alongside conventional clinical and biochemical assessment. Our findings correlate with the latest World Health Organization 2025 recommendations.

## Introduction

Tuberculosis (TB) continues to be a major public health burden across the world, and India continues to carry a disproportionate share of the global burden. The World Health Organization Global Tuberculosis Report 2025 states that India contributed to 25% of global TB cases in 2024 and 32% of global multidrug-resistant/rifampicin-resistant TB cases, even though TB incidence in the country declined by 21% from 2015 to 2024 [1]. Pleural TB is a common type of extrapulmonary manifestation of the disease and often poses a diagnostic challenge, particularly in high-incidence settings such as northern India.

Early recognition and appropriate treatment are important to reduce morbidity, mortality, and ongoing transmission. However, extrapulmonary TB is often difficult to confirm microbiologically because clinical specimens are typically paucibacillary. In tubercular pleural effusion (TPE), clinicians frequently depend on indirect evidence such as lymphocytic exudative pleural effusion, elevated adenosine deaminase (ADA), compatible symptoms including fever, cough, weight loss, chest pain, or breathlessness, supportive radiological findings, and response to antitubercular therapy (ATT).

This approach, although commonly used in practice, can create diagnostic uncertainty because pleural TB often yields very few organisms. Conventional Xpert *Mycobacterium tuberculosis*/rifampicin resistance (Xpert MTB/RIF) (Cepheid, Sunnyvale, CA) has a reported analytical detection threshold of about 113-131 colony-forming unit (CFU)/mL, whereas Xpert *Mycobacterium tuberculosis*/rifampicin resistance Ultra (Xpert MTB/RIF Ultra) (Cepheid) detects as few as about 15.6 CFU/mL [2], making it more suitable for paucibacillary specimens such as pleural fluid. Initiating ATT on clinical and biochemical grounds alone can also be questioned, since treatment is prolonged and may be associated with adverse effects such as hepatotoxicity, optic neuritis, hypersensitivity reactions, and gastrointestinal intolerance. Even with liquid culture systems such as mycobacterial growth indicator tube (MGIT), microbiological yield from pleural fluid remains low, and culture may take several weeks to become positive.

To address this limitation, molecular tests have been introduced, among which Xpert MTB/RIF Ultra has gained particular attention. Compared with conventional Xpert MTB/RIF, Ultra has a lower detection threshold and is designed to improve sensitivity in paucibacillary TB. A trace result on Ultra may reflect low-burden active disease, early infection, residual DNA, or occasionally a false-positive result [3]; therefore, it must be interpreted in the appropriate clinical context, especially when the pretest probability is high [4,5].

Although emerging studies suggest that Xpert MTB/RIF Ultra may improve detection in extrapulmonary and paucibacillary TB specimens, including pleural fluid, Indian real-world data remain limited. We therefore conducted an observational study in a retrospective manner at a tertiary care center in northern India to look for the diagnostic utility of Xpert MTB/RIF Ultra in presumed cases of TPE and to assess its role in rifampicin resistance determination.

Objectives

The primary objective of this study was to assess the diagnostic positivity rate of Xpert MTB/RIF Ultra in suspected TPE. The secondary objectives were to compare Xpert MTB/RIF Ultra positivity with MGIT culture positivity, to correlate Ultra positivity with clinical outcome and response to ATT, to evaluate the role of Ultra in rifampicin resistance determination, and, last but not least, to assess whether Ultra can be recommended as a routine investigation in suspected pleural TB.

## Materials and methods

This is an observational study done in a retrograde manner and was conducted at a tertiary care hospital in northern India over 18 months from July 2024 to December 2025. Patients with suspected TPE who underwent pleural fluid biochemistry, ADA testing, MGIT culture, and Xpert MTB/RIF Ultra testing were included. As per institutional protocol, pleural fluid collection was done under ultrasonography guidance with proper aseptic measures and collected in a sterile container. It was immediately transported to the in-house lab for processing. All patients were followed until completion of ATT. Serial chest X-rays were collected, including the chest X-ray at the end of treatment. Clinical improvements were documented.

Patients were managed by general physicians, pulmonologists, or both, according to institutional protocols and Indian national guidelines. Clinical records including demographic details, radiological findings, pleural fluid analysis, microbiological investigations, treatment details, and follow-up outcomes were reviewed retrospectively.

**Figure 1:**
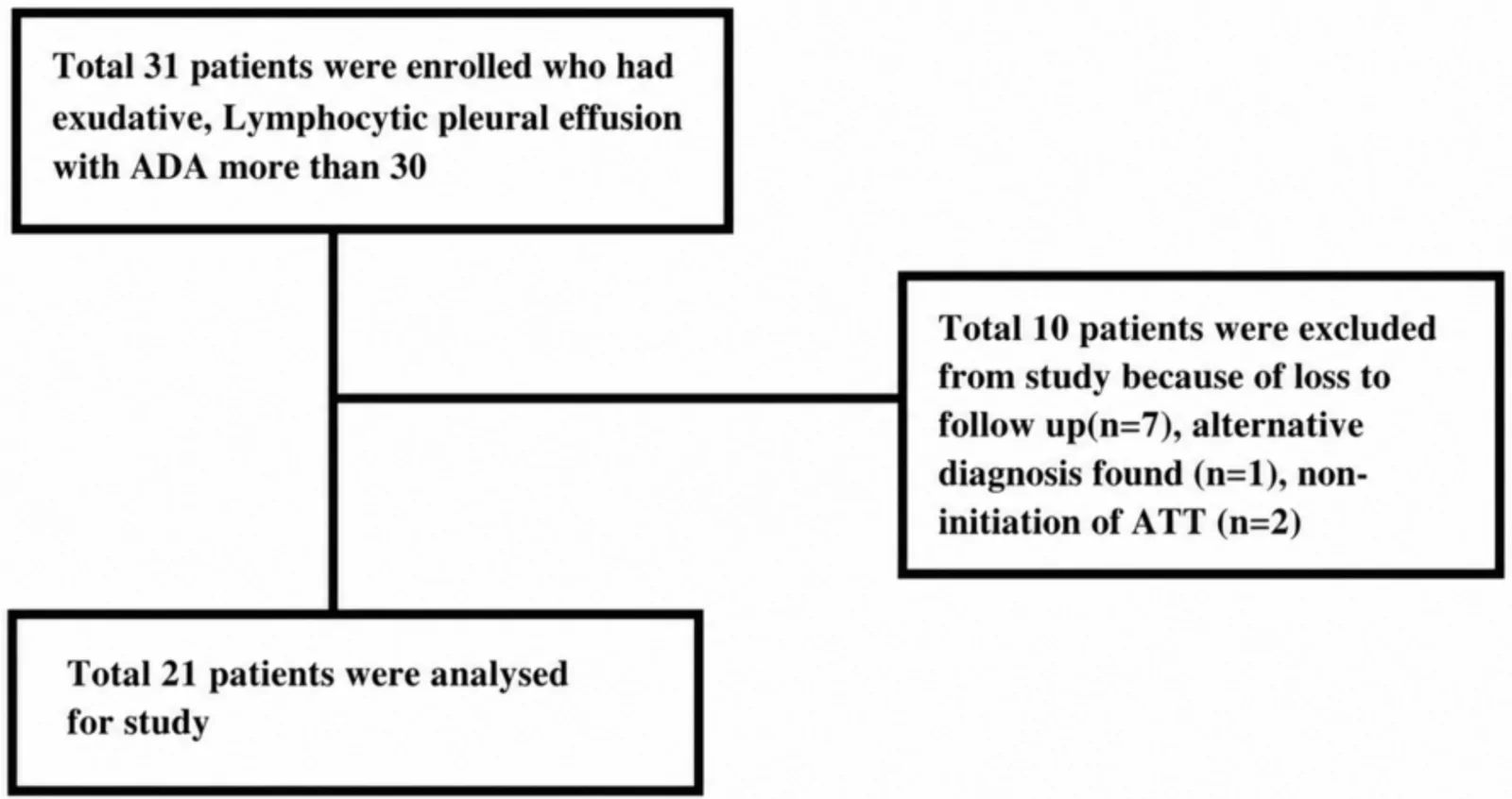
Data selection process ADA: adenosine deaminase; ATT: antitubercular therapy

A total of 31 patients with exudative (as per Light's criteria) lymphocytic pleural effusion, ADA levels greater than 30 IU/L, and clinicoradiological features suggestive of TB were screened (Figure 1). Of these, 21 patients who were started on ATT and completed follow-up were included in the final analysis. Ten patients were excluded because of loss to follow-up (n = 7), an alternative diagnosis (n = 1), or noninitiation of ATT (n = 2). All 10 excluded patients were negative for Xpert MTB/RIF Ultra. The collected data were entered into Microsoft Excel (Microsoft Corporation, Redmond, WA) and analyzed using Statistical Package for Social Sciences software, version 26 (IBM Corp., Armonk, NY).

Because pleural fluid culture is known to have limited sensitivity in pleural TB, due to the lower burden of tubercular bacteria, clinical response to ATT was also considered in the diagnostic correlation. Treatment response was included as an outcome measure because composite reference standards that combine microbiological tests with therapeutic response may better reflect true pleural TB in real-world practice [6].

## Results

A total of 21 patients were analyzed. Age ranged from 14 to 74 years, with a mean age of 30.62 years. Eighteen patients were younger than 40 years, while three were older than 40 years. Regarding sex distribution, there were 11 male and 10 female patients. Xpert MTB/RIF Ultra showed positive results in eight of 10 female (80%) and six of 11 male (54.5%) patients. Fisher's exact test was applied to find the significance level, and the results show a p value of 0.361 and an odds ratio (OR) of 0.30. This indicates there is no statistically significant association between sex and GeneXpert Ultra positivity (Table 1).

**Table 1:** Demographic profile with GeneXpert Ultra positivity and significance level (Fisher's exact test) OR: odds ratio

Parameter	Male	Female	p value	OR
Sex	11	10	0.361	0.30
GeneXpert Ultra positive	6	8		
Percentage of positivity	54.5	80	-	

**Figure 2:**
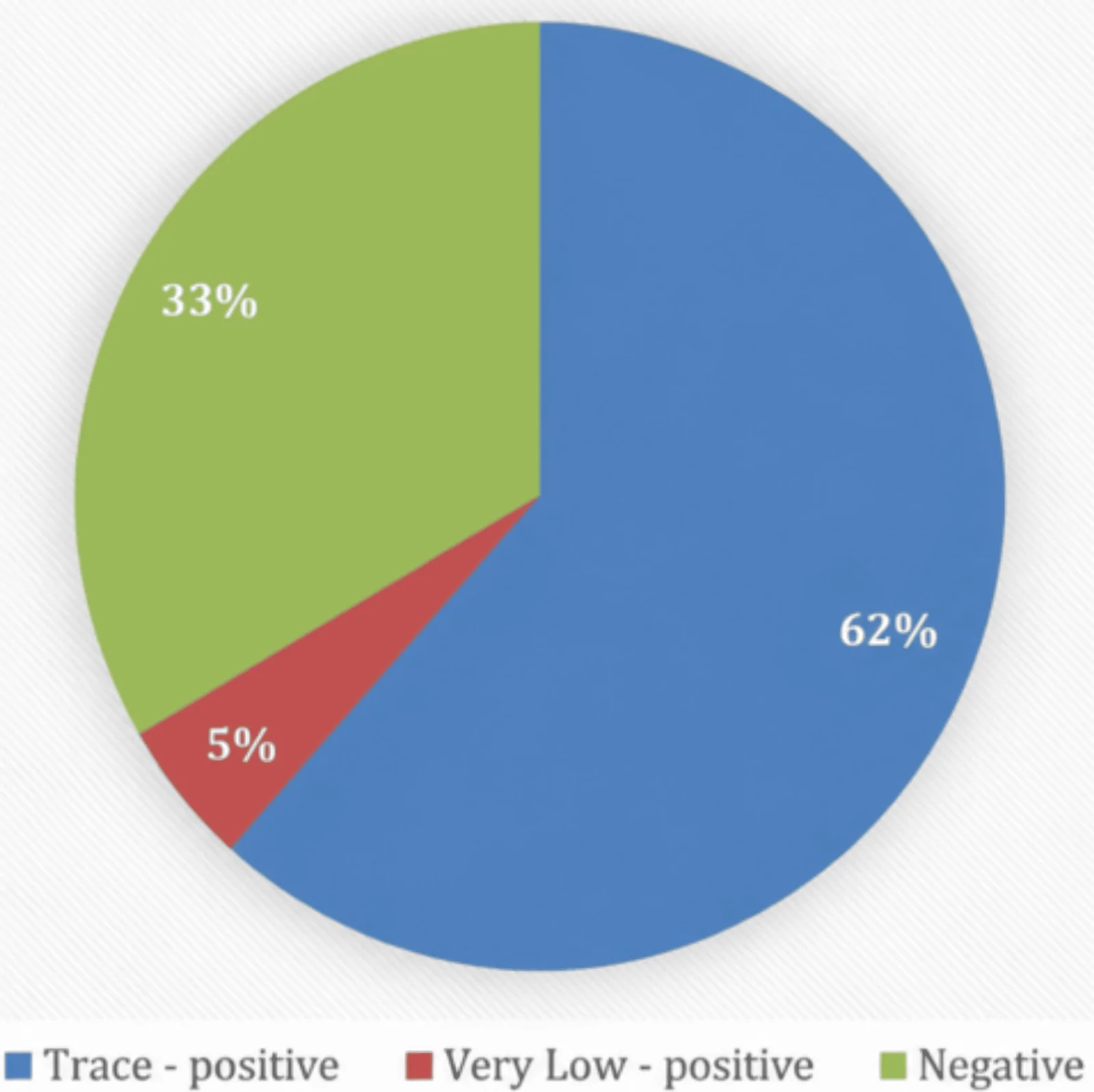
GeneXpert Ultra results

Overall, Xpert MTB/RIF Ultra showed positive results in 14 of 21 patients (66.7%), whereas MGIT culture remained negative in all cases. Among the Ultra-positive samples, 13 were reported as “trace” and one as “very low” (Figure 2).

As mentioned in the Methods section, to determine clinical response, we reviewed serial X-rays, including the X-ray at the end of treatment, and clinical parameters. All 21 patients showed clinical and radiological improvement (complete resolution of effusion and lung lesion ± presence of pleural thickening and fibrotic bands with complete resolution of cough, fever, and other clinical features) following ATT, supporting the diagnosis of pleural TB despite negative culture results.

A clear association was observed between the presence of coagulum in pleural fluid and Xpert MTB/RIF Ultra positivity. Coagulum was present in 13 of 14 Ultra-positive patients, compared with two of seven Ultra-negative patients. Fisher's exact test was applied to find the significance level, and the results show a p value of 0.0055 and an OR of 32.5. This indicates there is a statistically significant association between the presence of coagulum and GeneXpert Ultra positivity (Fisher's exact test, p = 0.0055). Patients with coagulum present had substantially higher odds of being GeneXpert Ultra positive (Table 2).

**Table 2:** Status of presence of coagulum in pleural fluid and significance level (Fisher's exact test) OR: odds ratio

Parameter	Coagulum present	Coagulum absent	p value	OR
Ultra positive (n1 = 14)	13	1	0.0055	32.5
Ultra negative (n2 = 7)	2	5		

These findings suggest that molecular amplification techniques may improve microbiological detection in paucibacillary specimens such as pleural fluid, which was our primary objective. In our study, Xpert MTB/RIF Ultra was positive in 14 of 21 patients (66.7%), whereas MGIT culture was negative in all 21 patients. Exact McNemar's test demonstrated that Xpert MTB/RIF Ultra detected significantly more positive cases than MGIT culture (14/21 (66.7%) vs. 0/21 (0%); p < 0.001).

Drug resistance determination

Xpert MTB/RIF Ultra also provides rifampicin resistance assessment; however, interpretation of resistance results in pleural fluid was limited. Thirteen of the 14 Ultra-positive samples showed indeterminate rifampicin resistance results, likely because trace positivity reflects an insufficient bacillary burden for reliable resistance interpretation. Among the study population, 13 patients had trace positivity on Ultra, and all of them showed indeterminate rifampicin resistance results. The single very low-positive sample showed rifampicin resistance not detected. Therefore, Ultra may be valuable for rapid TB detection; its role in rifampicin resistance determination in pleural fluid remains uncertain and requires validation in larger prospective studies.

## Discussion

TPE remains a diagnostic challenge, particularly in endemic and resource-limited settings. In routine practice, especially in northern India, clinicians often rely on elevated ADA, lymphocytic exudative pleural effusion, clinicoradiological findings, and therapeutic response when deciding to initiate ATT. However, the lack of microbiological confirmation frequently creates uncertainty, since ATT is prolonged and may be associated with significant toxicity.

Our study suggests that Xpert MTB/RIF Ultra may help bridge this diagnostic gap. The overall positivity rate in our series was 66.7%, which is comparable with previously published studies from outside India [6,7]. This finding is particularly relevant in clinically suspected cases with negative smear and culture results, where Ultra may provide useful microbiological support. We also observed that most Ultra-positive samples had coagulum in the pleural fluid, suggesting that simple biochemical and gross fluid characteristics may correlate with test positivity. In addition, the proportion of positive results was higher among female patients, although this observation should be interpreted cautiously because the sample size was small and the study was single-center.

The higher positivity rate with Xpert MTB/RIF Ultra compared with MGIT culture supports the role of molecular amplification assays in improving detection in paucibacillary pleural fluid specimens [6]. In our study, all Ultra-positive but culture-negative patients showed clinical and radiological improvement after ATT, which strengthens the clinical relevance of the Ultra findings. This aligns with previous evidence showing that molecular tests may outperform conventional culture in pleural TB where viable bacillary load is low.

Previous meta-analyses have also reported improved diagnostic performance of Xpert MTB/RIF Ultra over conventional Xpert MTB/RIF in pleural TB. Aggarwal et al. reported a pooled sensitivity of approximately 68% for Ultra compared with 52% for conventional Xpert, using culture-based reference standards [7]. Similarly, Hueda-Zavaleta et al. reported a sensitivity of 66.7% and specificity of 96.8% for Xpert assays in pleural fluid samples, highlighting the relatively high specificity but only moderate sensitivity of molecular testing in pleural TB [6].

However, rifampicin resistance determination remains a major limitation in pleural fluid specimens. Because many samples contain only trace bacillary load, resistance results may be indeterminate or unreliable. Therefore, while Ultra is a valuable tool for rapid diagnosis, its role in resistance detection in pleural effusion remains limited and should be interpreted with caution [8].

Limitations

The present study has many limitations. First, the sample size was small, and the data were derived from a single tertiary care center, which limits generalizability. Second, conventional Xpert MTB/RIF was not performed in all patients, so direct head-to-head comparison with Xpert MTB/RIF Ultra was not possible. Third, microbiological confirmation by culture remained limited because pleural TB is typically paucibacillary. However, clinical and radiological response to ATT was considered an additional supportive indicator in the diagnostic assessment.

## Conclusions

In endemic and resource-limited settings, diagnosis of TPE continues to depend largely on clinical judgment and pleural fluid biochemistry because microbiological confirmation is often difficult. In our study, Xpert MTB/RIF Ultra showed a significant diagnostic yield in suspected pleural TB and correlated well with clinical response to ATT, even in MGIT-negative patients. However, rifampicin resistance interpretation remains limited in paucibacillary pleural fluid specimens.

Xpert MTB/RIF Ultra appears to be a useful adjunctive diagnostic tool. Based on these findings, it may be considered alongside conventional clinical and biochemical assessment in suspected TPE. We recommend that Xpert MTB/RIF Ultra be done in all suspected TPE cases as a routine test. It carries more significance in endemic zones like India.
